# Polypyrimidine Tract Binding Protein Functions as a Negative Regulator of Feline Calicivirus Translation

**DOI:** 10.1371/journal.pone.0009562

**Published:** 2010-03-10

**Authors:** Ioannis Karakasiliotis, Surender Vashist, Dalan Bailey, Eugenio J. Abente, Kim Y. Green, Lisa O. Roberts, Stanislav V. Sosnovtsev, Ian G. Goodfellow

**Affiliations:** 1 Department of Virology, Imperial College London, London, United Kingdom; 2 Laboratory of Infectious Diseases, National Institute of Allergy and Infectious Diseases, National Institutes of Health, Bethesda, Maryland, United States of America; 3 Department of Cell Biology and Molecular Genetics, University of Maryland, College Park, Maryland, United States of America; 4 Faculty of Health and Medical Sciences, University of Surrey, Guildford, United Kingdom; Yonsei University, Republic of Korea

## Abstract

**Background:**

Positive strand RNA viruses rely heavily on host cell RNA binding proteins for various aspects of their life cycle. Such proteins interact with sequences usually present at the 5′ or 3′ extremities of the viral RNA genome, to regulate viral translation and/or replication. We have previously reported that the well characterized host RNA binding protein polypyrimidine tract binding protein (PTB) interacts with the 5′end of the feline calicivirus (FCV) genomic and subgenomic RNAs, playing a role in the FCV life cycle.

**Principal Findings:**

We have demonstrated that PTB interacts with at least two binding sites within the 5′end of the FCV genome. *In vitro* translation indicated that PTB may function as a negative regulator of FCV translation and this was subsequently confirmed as the translation of the viral subgenomic RNA in PTB siRNA treated cells was stimulated under conditions in which RNA replication could not occur. We also observed that PTB redistributes from the nucleus to the cytoplasm during FCV infection, partially localizing to viral replication complexes, suggesting that PTB binding may be involved in the switch from translation to replication. Reverse genetics studies demonstrated that synonymous mutations in the PTB binding sites result in a cell-type specific defect in FCV replication.

**Conclusions:**

Our data indicates that PTB may function to negatively regulate FCV translation initiation. To reconcile this with efficient virus replication in cells, we propose a putative model for the function of PTB in the FCV life cycle. It is possible that during the early stages of infection, viral RNA is translated in the absence of PTB, however, as the levels of viral proteins increase, the nuclear-cytoplasmic shuttling of PTB is altered, increasing the cytoplasmic levels of PTB, inhibiting viral translation. Whether PTB acts directly to repress translation initiation or via the recruitment of other factors remains to be determined but this may contribute to the stimulation of viral RNA replication via clearance of ribosomes from viral RNA.

## Introduction

The regulation of mRNA translation by RNA-binding proteins is an essential mechanism for the control of gene expression. RNA-binding proteins form functional ribonucleoprotein complexes (RNPs) that determine the fate of the cognate mRNAs. They have been shown to play crucial roles in RNA metabolism, such as pre-mRNA splicing, capping and polyadenylation, tRNA maturation, mRNA localization and translation, which in turn affect the vast majority of cellular processes.

Polypyrimidine tract binding (PTB) protein, one of the best studied RNA-binding proteins, is a 57 kDa protein with four RNA recognition motifs (RRMs) and an affinity for pyrimidine-rich RNA sequences [1]. The interaction of the individual RRMs with different sites on the same RNA, often distant in terms of their nucleotide position in the primary sequence, can result in substantial restructuring of the RNA in order to adopt a “functional conformation” [2], [3]. This RNA chaperone activity of PTB is well documented and is known to be important for the life cycle of many viruses [4]. PTB has a primarily nuclear localization but is has been demonstrated that specific signals, such as PKA phosphorylation [5], viral infections [6] or alterations in nuclear pore permeability [7], may lead to its translocation to the cytoplasm. Nuclear PTB is a negative regulator of pre-mRNA alternative splicing [8] and in some cases regulates mRNA polyadenylation [9]. Cytoplasmic PTB affects α-actin mRNA localisation in neurites, while the *Xenopus* homologue of PTB regulates Vg1 mRNA localisation [10]. One extensively studied role of cytoplasmic PTB is in the regulation of IRES-dependent translation of several cellular and viral mRNAs. PTB is required for the IRES-dependent translation of Bag-1, Apaf-1 and p53 mRNAs [2], [3], [11] and it is also required for the efficient function of the poliovirus (PV) [12], foot-and-mouth disease virus (FMDV) [13] and Theiler's murine encephalomyelitis virus (TMEV) [14] IRES elements. A context-dependent requirement of PTB has also been described for encephalomyocarditis virus (EMCV) translation [15]. While a role for PTB in HCV IRES function has been proposed, the exact function in this context is still rather unclear [16], [17], [18]. In some cases (e.g. Dengue virus) PTB regulates viral replication rather than translation [19], [20]. Using translation-deficient defective interfering murine hepatitis virus (MHV) RNAs it was also shown that PTB is required for MHV replication [21].

**Figure 1:**
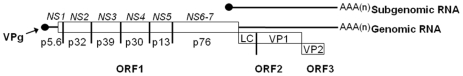
Schematic representation of the feline calicivirus genome. Diagrammatic illustration of the three open reading frames present within the feline calicivirus genome. The mature components of ORF1 are highlighted along with the recently proposed NS1-7 nomenclature used for murine norovirus [64]. The positions of the leader of the capsid protein (LC), as well as the major and minor capsid proteins (VP1 and VP2) are also highlighted.

The *Caliciviridae* family of positive-strand RNA viruses is divided into four currently defined genera: *Norovirus*, *Sapovirus*, *Lagovirus* and *Vesivirus*. Human caliciviruses (HuCV), from the *Norovirus* and *Sapovirus* genera are a major cause of acute gastroenteritis and are responsible for more than 85% of non-bacterial gastroenteritis outbreaks in Europe [22]. Despite recent advances no suitable and efficient cell culture system is, as yet, available for the propagation of HuCVs *in vitro* [23], [24], [25], [26]. In contrast, feline calicivirus (FCV) and murine norovirus (MNV) can be efficiently propagated in conventional tissue culture and are readily used as models for the study of molecular mechanisms that regulate the life cycle of caliciviruses [27]. The FCV genome of 3 open reading frames (ORFs, Fig 1); ORF1 is translated from the genomic RNA (gRNA) producing the non-structural proteins (NS1, NS2, NS3, NS4, NS5 and NS6-7), while ORF2 and ORF3, encoding the major and minor capsid proteins (VP1 and VP2 respectively) are expressed from a subgenomic RNA (sgRNA) produced during infection [28].

Calicivirus protein synthesis is thought to occur via a unique mechanism not found in any other animal RNA viruses. This mechanism relies on ribosomal recruitment by the VPg (NS5) protein, which is covalently linked to the 5′ end of the viral RNA, through the interaction of VPg with various translation initiation factors [29], [30], [31], [32], [33]. During our previous studies we demonstrated a specific interaction of PTB with the 5′extremity of the FCV gRNA and sgRNA, and also the requirement for PTB in the FCV replication cycle [34]. In the present report we have further characterized the interaction between PTB and the FCV genome, identifying the binding site of PTB at the 5′ end of the gRNA and demonstrating that PTB functions as a repressor of FCV translation. We believe this represents a new mechanism to promote the switch from translation to replication of a positive-strand RNA virus and therefore represents a novel function for the PTB protein.

## Materials and Methods

### Materials

FCV, strain Urbana, was generated by transfection of RNA transcripts derived from the full-length infectious clone pQ14 [35] into Crandell–Reese feline kidney (CRFK) cells obtained from ATCC. Antisera to the FCV RNA polymerase NS6-7 (p76) were produced as described in Karakasiliotis et al. 2006 [34]. Antisera for western blot to hnRNP I (PTB) was purchased from Santa Cruz Biotechnology, mouse monoclonal antibodies to PTB DH7 and DH17 were kindly provided by Eckard Wimmer (Stony Brook University, New York, USA). Antiserum to GAPDH was purchased from Ambion. Antiserum to FCV Urbana capsid (VP1) was as previously described [35] .

### Protein Purification

For the purification of his-PTB SG13009 *E. coli* cells were transformed with a pQE9 plasmid expressing his-PTB1 provided by Stephen Curry (Imperial College, London). The protein purification was performed using Ni-NTA (Qiagen) according to the manufacturer's instructions. His-PTB was dialyzed against 50 mM HEPES pH 7.6, 1 mM DTT, 1 mM MgCl2 and 20% glycerol. GST-PTB was purified from DH5α *E. coli* cells transformed with the pGEX6P-1 PTB plasmid, provided by Richard Jackson (University of Cambridge, UK). Cells were grown in LB at to an OD600 of 0.6, isopropyl-β-D-thiogalactopyranoside (IPTG) was added (0.1 mM final) and the protein expression was allowed to occur for 2 hours. The cells were then lysed using a French press in PBS containing 0.5 mM DTT, 10 mM EDTA, 3 µg/ml leupeptine and pepstatin, 1% Triton X-100, 1 mM PMSF. GST-PTB was then purified from the clarified lysate using glutathione-sepharose, washing with PBS containing 0.5 mM DTT, 10 mM EDTA and 1% Triton X-100. A second wash with 50 mM Tris pH 8.0, 150 mM NaCl and 0.5 mM DTT followed and the protein then eluted in 10 mM reduced glutathione, 50 mM Tris pH 8.0, 150 mM NaCl and 0.5 mM DTT. Eluted protein was dialyzed against 50 mM HEPES pH 7.6, 1 mM DTT, 1 mM MgCl2 and 20% glycerol and stored at −80°C until further use.

### Mutagenesis and Reverse Genetics Recovery

Mutagenic oligonucleotides were synthesized and used for site directed mutagenesis of the full-length infectious clone pQ14 [35] (primer details available upon request). Generated cDNA constructs were sequenced in their entirety prior to virus recovery. Wild type and mutant viruses were recovered by transfection of CRFK cells with capped, *in vitro* transcribed RNA derived from the full-length cDNA clones using Lipofectamine 2000 (Invitrogen). High titre virus stocks were prepared by an additional passage in CRFK cells and viral titers were determined by TCID50. RT-PCR was used to confirm the virus stocks retained the introduced mutations.

### Growth Kinetics Analysis

Growth curves of wild type and mutant virus (mBS2+3) were performed in AK-D (feline fetal lung, obtained from ATCC), CRFK (feline kidney cell line, obtained from ATCC) and FEA (feline embryonic airway, obtained from Alan Radford, University of Liverpool) cells to assess viral growth kinetics. Cells were plated in 24 well plates at a density of 2×10^5^cells/well. Single step growth curves were performed using an initial multiplicity of infection (m.o.i.) of 3 TCID50 per cell, based on the titre in CRFK cells. Samples were taken in triplicate for TCID-50 titration at 0, 4, 6, 8, 10 and 12 hours post infection (hpi). Multi-step growth curve analysis was performed using a m.o.i. of 0.01 and harvesting at 0, 4, 8, 12, 24 and 28 hpi.

### *In Vitro* Translation Assays

FCV VPg-linked RNA was purified from FCV replication complexes isolated from FCV infected CRFK cells as described previously [31]. The 5′cap-CAT-FMDV IRES-Luc dicistronic RNA was produced by T7 *in vitro* transcription from the dicistronic plasmid pGEM-CAT/FMDV/LUC [36]. The dicistronic RNA was polyadenylated using *E.coli* Poly(A) polymerase (Ambion). The FCV 5′G_1–245_ RNA was produced by PCR using primers which introduced a T7 RNA polymerase promoter at the 5′ end of the PCR product (details available upon request) followed by *in vitro* transcription. The *in vitro* translation assays were performed in Flexi rabbit reticulocyte lysates (RRLs) (Promega). Each 12.5 µl translation reaction included 6.25 µl of RRL, 20 µM amino acid mixture minus methionine, 100 mM KCl, 2 mM DTT, 0.5 mM MgOAc, 5 µCi ^35^S methionine (1000 Ci/mmol) (GE Healthcare) and 312.5 ng of an RNA preparation containing FCV VPg-linked RNA or *in vitro* transcribed dicistronic RNA. Translation reactions occasionally contained 1 µl of his-PTB at various concentrations, and 5′G_1-245_ RNA transcripts at various concentrations. The reactions were incubated at 30°C for 1.5 h.

### Biochemical Structure Probing

Biochemical RNA structure probing was performed using the conformational specific RNases V1, A, T1 (Ambion) and T2 (MoBiTec GMBH). *In vitro* transcribed, gel purified and refolded RNA (1 µg) was mixed with yeast RNA (1 µg) and structure buffer provided with the RNases (Ambion). Enzymatic cleavage reactions were performed at 30°C for 10 minutes with enzyme concentrations empirically determined to generate single cleavage products. The reactions were stopped using inactivation/precipitation buffer, the RNA precipitated, subsequently washed with 70% ethanol and resuspended in 10 µl of nuclease free water. An aliquot of the digested RNA (2 µl) was then used for primer extension with end labeled primers and AMV reverse transcriptase as described by the manufacturer (Promega). A sequencing ladder generated using the same primer was run along with the primer extension products on a 7M urea 6% acrylamide sequencing PAGE. The gel was dried and exposed to a phosphorimager screen.

### Electrophoretic Mobility Shift (EMSA) Assays

Radioactive probes were generated by *in vitro* transcription of PCR products containing the region of interest under the control of a T7 RNA polymerase promoter (primer details available upon request). Radioactively labeled RNA was subsequently gel purified by denaturing urea PAGE, the probe excised, eluted and precipitated prior to quantification by spectrophotometry. RNA was refolded by heat denaturation followed by slow annealing (∼1°C/min) prior to use. Typically a 7.5 µl EMSA reaction contained 85 nM radioactive RNA probe, GST-PTB at various concentrations (see figures for more details), 0.67 µg/µl yeast tRNA (Ambion), 50 mM HEPES pH 7.6, 25 mM KCl, 2.5 mM MgCl2, 1 mM DTT and 4% glycerol. All reactions were incubated for 10 min at 30°C and the reactions analyzed on a 4% acrylamide gel (acrylamide:bis-acrylamide 19:1) containing 5% glycerol. The EMSA gels were dried and visualized on a phosphorimager screen. Band quantification was performed using the ImageQuant 5.0 software package (Molecular Dynamics) and the amount of protein required to shift 50% of the probe was determined using SigmaPlot. Note that relative, rather than absolute, affinity was determined due to the difficulty in determining the active fraction of the purified GST-PTB.

### RNase H Mediated Inactivation of VPg-Linked FCV Genomic RNA

The specific digestion of the FCV genomic RNA in the viral RNA preparation from infected cells was carried out using RNase H and an oligonucleotide that is complementary to NS6-7 coding region (ntds positions 3215-3244). RNase H reactions contained 1.26 µg of VPg-linked RNA preparation, 25 pmol of primer, 10 U RNAsin (Promega), 37.5 mM KCl, 25 mM Tris-HCl pH 8.3, 1.5 mM MgCl2, 5 mM DTT and 50 U of RNase H (Ambion). The reaction was incubated at 37°C for 20 min. M13/Puc R was used as non-specific control oligonucleotide to control for any effects of RNase treatment and subsequent extraction on the replication/translation of the viral RNA.

### Confocal Microscopy Analysis of PTB Localization

CRFK cells were plated on round glass cover slips and infected with FCV (m.o.i. of 0.5) for 16 h at 32°C . The glass cover slips were washed with chilled PBS and fixed in 4% paraformaldehyde solution containing 250 mM HEPES pH 7.4 for 10 min on ice. An 8% paraformaldehyde solution containing 250 mM HEPES pH 7.4 was applied for 20 min at room temperature and the cells were washed 3 times in PBS. 50 mM ammonium chloride was applied for 10 min at room temperature and the cells were washed 3 times in PBS. Permeabilization of the cells was achieved using 0.2% Triton-X 100 in PBS for 10 min at room temperature followed by three 5-minute washes in block buffer (1% bovine serum albumin in PBS). NS6-7 staining was performed using a rabbit polyclonal antibody and PTB staining was performed using a mouse monoclonal antibody for PTB (DH17). Image-iT FX signal enhancer was applied according to the manufacturer's instructions (Invitrogen). α-rabbit Alexa Fluor 488 (Invitrogen) and α-mouse Alexa Fluor 546 (Invitrogen) were used as secondary antibodies. The cover slips were mounted on a glass slide with 40 µl of Mowiol 4–88 (Calbiochem) containing 1 µg/ml of DAPI. The cells were viewed under a Zeiss LSM 510 Meta confocal microscope, and the images were processed with Zeiss Meta 510 software. For the quantification of staining intensity in specific areas of the confocal images Image Quant 5.0 software (Molecular Dynamics) was used as described previously [37]. To examine the steady state localization of PTB in the FEA, CRFK and AK-D cells, cells were plated, left untreated then subsequently fixed and stained as described above, except that goat α-mouse Alexa Fluor 488 (Invitrogen) was used to detect binding of the PTB monoclonal antibody. Live time-lapse fluorescence microscopy was employed to monitor changes in the PTB protein localization in FCV-infected CRFK cells. CRFK cells (2×10^6^) grown on glass chamber slides were transfected with the pEGFP-PTB (kindly provided by Chris Smith, Cambridge) or a combination of pEGFP-PTB and pDsRed2-Nuc (Clontech). 24 hrs post transfection, cells were then infected with either FCV-LC-DsRed [38] or with FCV Urbana using an m.o.i. of 2. The inoculated cells were then transferred to an environmental chamber (37°C and 5% CO2), and time-lapse imaging was performed using a Leica SP5 X-WLL confocal microscope (Leica, Germany). Images were taken every five minutes, with channel (GFP and DsRed2) data collected independently. Sequences of the confocal images were transformed into time-lapse movies using Imaris software (version 6.4, Bitplane AG, Zurich, Switzerland).

### RNA Immunoprecipitation

10^7^ CRFK cells were infected with FCV for 6 h using an m.o.i. of 0.1. Ribonucleoprotein complexes were extracted from FCV infected CRFK cells and subjected to immunoprecipitation following the previously described procedure [39]. DH7 and DH17 mouse monoclonal antibodies were used for the immunoprecipitation of the RNAs bound to PTB. NS6-7 and GAPDH specific antisera were used as positive and negative controls respectively.

## Results

### Biochemical Structure Determination of the 5′ Extremity of the FCV Genome

**Figure 2:**
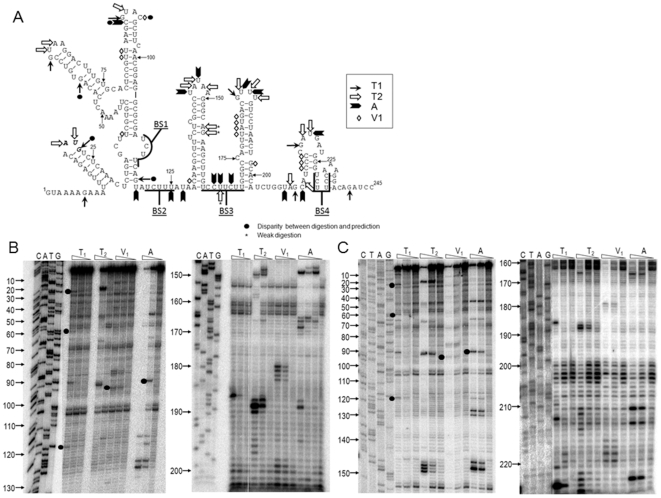
Sequence and structure of the 5′ end of the feline calicivirus genome. (A) RNA sequence of nucleotides 1-245 of the feline calicivirus genomic RNA, highlighting the four potential PTB binding sites (BS1-4). Note that the pyrimidine rich sequences around the core PTB binding sequence (UCUU) are highlighted. The ORF1 AUG initiation codon at position 20 is shown in bold italics. The positions of single strand specific RNases T1,T2 and A, as well as the double-strand specific RNase V1, obtained from RNase sensitivity mapping (shown in panel B and C) are highlighted as detailed in the symbol legend. (B) *In vitro* transcribed RNA representing nucleotides 1-245 of FCV genomic RNA was subjected to limited RNase digestion and primer extension analysis using either a primer binding between nucleotides 223 to 204 (B) or nucleotides 245–228 (C). A sequencing ladder was generated using same primers and run on the gel along with primer extension products to identify the cleavage sites of RNases. The positions of RNase cleavage sites which show disparity with the predicted RNA structure are highlights with a black filled circle. Note that only cleavage sites which were apparent in multiple experiments are depicted.

During our previous analysis of the interaction of PTB with the FCV genome, we demonstrated that bacterially expressed recombinant GST tagged PTB can bind in a sequence specific manner to nucleotides (ntds) 1-245 and 1-284 of the 5′ ends of the FCV gRNA and sgRNA, respectively [34]. Sequence analysis demonstrated that the 5′ end of the gRNA contains 4 regions which match the PTB binding site consensus previously identified (UCUU) [40], [41] (BS1-4 in Fig 2a). To better define the role of PTB in the FCV life cycle and to allow the generation of mutations which did not result in large scale disruption of the RNA secondary structure, we first determined the structure of the 5′ end of the FCV genomic RNA using ribonuclease (RNase) digestion. This analysis uses RNases specific for either single-stranded (T1, T2 or A) or double-stranded (V1) RNA to determine the solution structure of a particular transcript. This type of analysis has to date, not been undertaken on the 5′ end of a genome for any member of the *Caliciviridae* family. RNase cleavage sites were determined using primer extension with two different primers (Fig 2b and c) and then mapped onto a structure predicted using the MFold software [42] (Fig 2a). Clearly defined regions of single- and double-stranded RNA were observed and in the majority of cases the predicted structure was confirmed. However a limited number of regions showed some disparity (highlighted in Fig 2a); for example G57 is predicted to form a G-U interaction with U72, however a clear T1 RNase cleavage site was obtained. In addition C93 is predicted to lay within a single-stranded loop sequence, and indeed positions G90 and U91 were found to be single-stranded, however clear RNase V1 cleavage was obtained at position 93 (Fig 2b and c). The positions displaying disparity are likely the result of flexibility or “breathing” often observed in RNA structures and/or additional tertiary RNA-RNA interactions which occur and which are not detected using standard structure predictions.

### PTB Binds to at Least Two Sites in the 5′ End of the FCV Genomic RNA

**Figure 3:**
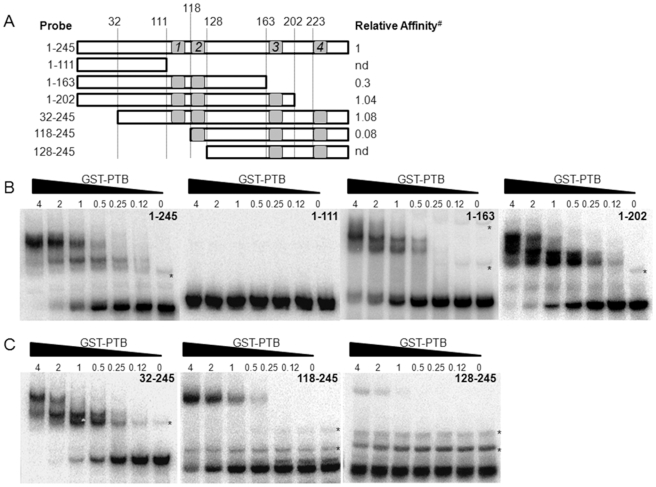
Identification of PTB binding sites using truncation analysis. EMSA analysis of GST-PTB binding to probes encompassing various regions of the FCV 5′ end. Radiolabeled RNA probes were generated by *in vitro* transcription of PCR products encompassing the various regions of the FCV genome, followed by gel purification. EMSA reactions were set up as described in the text and the reactions analyzed by native PAGE. Gels were then dried, exposed to phosphorimager screen and the amount of probe in a complex with PTB determined using ImageQuant software. (A) Diagrammatic representation of the truncations under study highlighting the relative affinity of PTB for the transcript. # highlights that the affinity is expressed relative to WT 1–245 probe (set at 1). nd denotes that the relative affinity could not be determined due to insufficient complex formation. EMSA gels using the 1–245 transcript along with the 3′ end truncations (B) or 5′ end truncations (C). EMSAs were performed a minimum of three times and the data obtained from one representative experiment shown. Asterisks are used to highlight the position of alternative conformations of the RNA probe which make up a minor component of the refolded RNA transcript.

To further characterize the interaction between PTB and the 5′ end of the FCV gRNA, truncations of the region were used as radiolabeled probes for EMSA analysis (Fig 3). The predicted PTB consensus binding sequences (BS1-BS4, Fig 2a) were taken into consideration during the design of the truncations (Fig 3a) in order to analyze the relative contribution of the potential binding sites to PTB binding. Also, to minimize the effect of RNA truncations on the overall folding of the RNA transcripts, the secondary structure of the region, as determined biochemically (Fig 2a) was also taken into account. Two sets of truncations were tested for their ability to form a complex with GST tagged PTB by EMSA analysis. The FCV 5′ end probe encompassing ntds 1-245 was truncated at the 3′ end producing RNA transcripts containing ntds 1–111, 1–163 and 1–202 as illustrated in Fig 3a. Ntds 1–111 did not contain any of the PTB consensus sequences, 1–163 encompassed the potential binding sites BS1 and BS2, with the 1–202 sequence containing the BS1, BS2 and BS3 consensus sequences. Truncations from the 5′ end were also generated to produce RNA transcripts that encompassed ntds 32–245, 118–245 and 128–245 (Fig 3a). Ntds 32–245 contained all predicted PTB consensus sequences, but lacked the first stem loop at the 5′ end, nts 118–245 encompassed BS2, BS3 and BS4 potential binding sites and ntds 128–245 contained BS3 and BS4 consensus sequences (Fig 2a). Bioinformatic analysis indicated that the truncations did not have any detrimental effect to the overall structure adopted by the RNA or the structural context of the potential PTB binding sites (data not shown).

As expected, PTB was unable to bind to the transcript 1–111, containing none of the predicted PTB binding sites (Fig 3a and b). Addition of the first two potential binding sites (BS1 and BS2), in the transcript 1–163, dramatically increased the ability of PTB to bind (Fig 3a and b). The further addition of BS3, using the transcript 1–202, restored the relative affinity of the RNA for PTB to the levels observed using ntds 1–245 level (Fig 3a and b). Deletion of the first 31 ntds from the 5′ end of the 1–245 transcript, which removes the entire first stem loop (Fig 2a) producing the transcript 32–245, did not disrupt PTB binding whereas the deletion of the first 117 ntds, removing BS1 (as in the 118–245 probe), led to a significant reduction in PTB binding (Fig 3a and c). The additional deletion of BS2, producing the transcript 128–245, resulted in the almost complete loss of PTB binding (Fig 3a and c). These data indicated that sequences around BS1, BS2 and BS3 contribute to PTB binding (compare binding to 118–245 and 1–163 with 1–202, Fig 3a and c). It was evident that “spatial context” in which BS2 and 3 are present also plays a role as the transcript 118–245, containing both BS2 and 3 displayed a reduced ability to bind PTB (compare 118–245 with 1–245 Fig 3a and c).

### Mutation of BS2 and BS3 Reduces PTB Binding to the 5′ End of the FCV Genome

**Figure 4:**
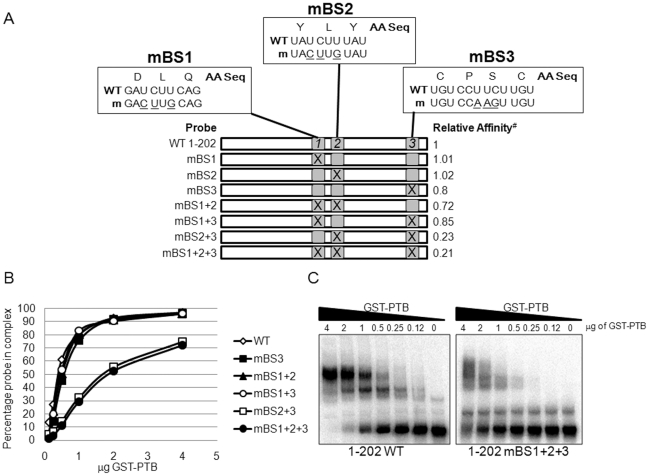
Identification of PTB binding sites using synonymous mutations. EMSA analysis of GST-PTB binding to wild type (WT) or mutant derivatives of nucleotides 1–202 of the FCV 5′ end. Radiolabeled RNA probes were generated by *in vitro* transcription of PCR products, followed by gel purification. EMSA reactions were set up as described in the text and the reactions analyzed by native PAGE. Gels were then dried, exposed to phosphorimager screen and the amount of probe in a complex with PTB determined using ImageQuant software. (A) Diagrammatic representation of the region under study highlighting the binding sites (BS1-3), the mutations introduced (m) are underlined and the amino acid sequence coded by that region of the genome is also shown. The relative affinity of PTB for the transcript is shown based on quantification of PTB binding. # highlights that the affinity is expressed relative to WT 1–202 probe. (B) Quantification of PTB binding to WT or PTB binding site mutants (mBS1-3). The amount of radiolabeled probe in a complex with PTB was determined using phosphoimager and expressed as a percentage of the total. Note that for clarity only data for the single binding site mutant BS3 (mBS3) is shown but mBS1 and mBS2 displayed identical binding curves (data not shown). (C) Representative EMSA gel displaying the binding of GST-PTB to WT RNA probe or a probe containing synonymous changes in binding sites 1, 2 and 3 (mBS1+2+3). EMSAs were performed a minimum of three times and the data obtained from one representative experiment shown.

To clarify the contribution of the other potential binding sites, mutations were introduced in order to disrupt the PTB consensus binding sequence in BS1, BS2 and BS3 in all possible combinations (Fig 4a). As all the potential binding sites were located in the protein-coding region, the mutations were designed to preserve the original amino-acid sequence (synonymous nucleotide changes) while altering the pyrimidine rich sequence in order to allow subsequent analysis of their effects on virus replication (detailed below). All mutations were generated in a probe encompassing ntds1–202 as our data indicated that this probe contained all the necessary sequences required for efficient PTB binding (Fig 3). The mutation of individual binding sites showed no effect on PTB binding (data not shown, Fig 4a and b). The triple binding site mutant mBS1+2+3, containing synonymous changes in all three potential binding sites, showed a dramatic reduction in PTB binding (Fig 4) while the only double mutant that demonstrated similar defects in PTB binding was mBS2+3 (Fig 4a and b). As the PTB binding curve for the mBS2+3 mutant was the same as the triple mutant, it was concluded that the minimum combination of mutations required to reduce PTB binding were in BS2 and 3. Clearly, although mutation of BS2 and 3 reduced PTB binding, some residual binding activity remained (Fig 4). This suggested that either additional sequences contributed to PTB binding or that the introduced synonymous changes, which do not remove all pyrimidines within the binding sites, are not sufficient to completely disrupt PTB binding. To address this, the mutational analysis was extended using RNAs in which the polypyrimidine tracts of BS1, 2 and 3 were fully substituted for adenines (full(A) mutant). PTB binding of the full(A) mutant RNAs was identical to that observed for the synonymous mutations, showing that the residual binding observed for the synonymous mutant RNAs was not due to remaining pyrimidines in the potential binding sites (data not shown).

### PTB Translocates from the Nucleus to the Cytoplasm during FCV Infection

**Figure 5:**
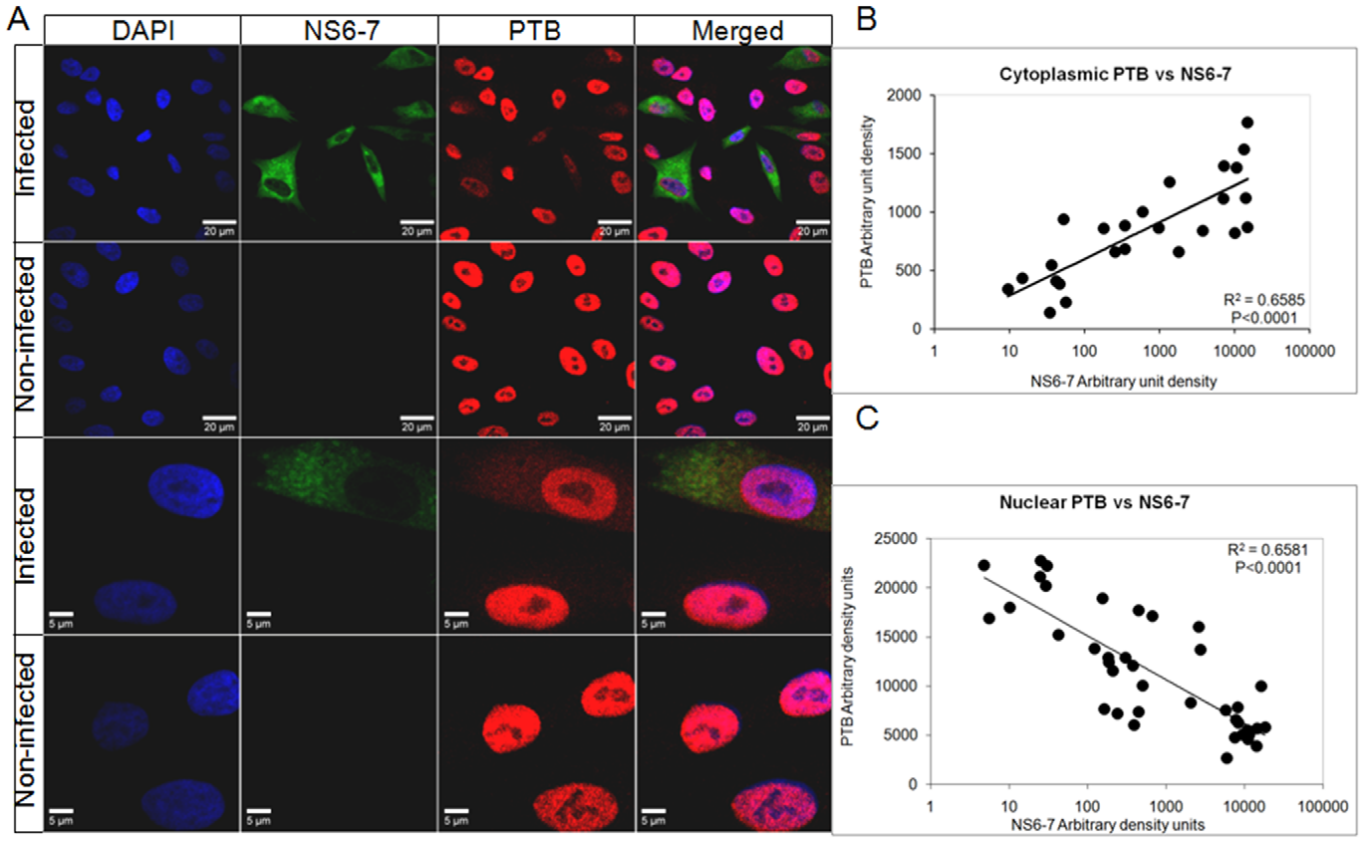
Feline calicivirus infection results in the redistribution of PTB. CRFK cells were infected with FCV for 16 h at 32°C and at an m.o.i. of 0.5 (A) Infected and control cells were fixed and stained for NS6-7 (green) and PTB (DH17 monoclonal, red). DAPI was used for nuclei (blue) staining. The cells were observed using confocal microscopy. Quantification of nuclear (B) and cytoplasmic (C) PTB density (arbitrary intensity units/arbitrary volume units) against the density of NS6-7 in the cytoplasm of the same cell. Individual NS6-7 expressing cells were selected and a region of interest selected in the cytoplasm and nucleus. The levels of NS6-7 staining (green) and PTB staining (red) was quantified in arbitrary units and plotted accordingly (see materials and methods for further details).

**Figure 6:**
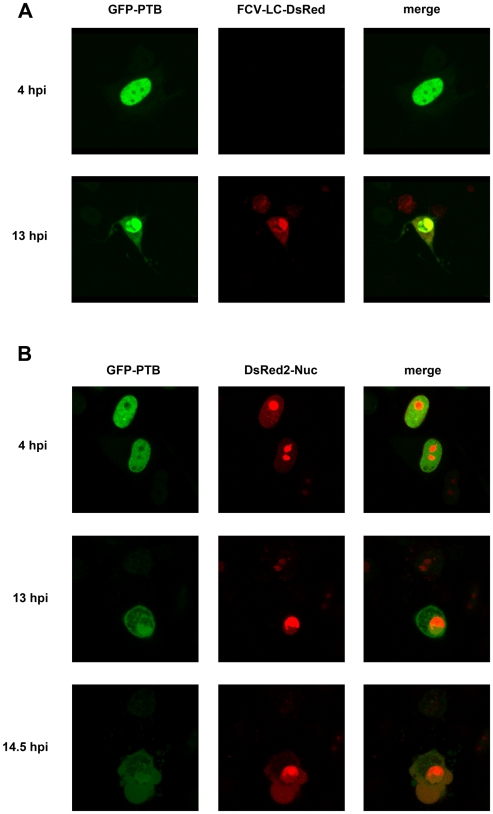
Feline calicivirus infection causes both PTB specific and general effects on nuclear localization of proteins. (A). GFP-PTB expressing CRFK cells were infected with recombinant FCV expressing a LC-dsRED fusion protein (m.o.i. 2) and examined during the course of the infection. (B) Cells transfected with cDNA constructs expressing PTB-GFP and dsRED fused to 3 copies of the nuclear localization sequence of the SV40 large T-antigen (DsRED2-Nuc) were infected with FCV at a m.o.i. of 2. Cells were then examined over the course of the infection. See supplementary files Figure S1 and Figure S2 for AVI data relating to A and B respectively.

To begin to determine the role that PTB plays in the FCV life cycle we first examined how virus infection altered PTB localization. Infected CRFK cells were stained for PTB and the viral polymerase, NS6-7, as a marker for the viral replication complexes. The localization of PTB in uninfected cells is predominantly nuclear (Fig 5a). We observed that during FCV infection of CRFK cells at 37°C PTB remained predominantly nuclear at all stages of infection, while only a small fraction of infected cells, typically those expressing high levels of NS6-7, demonstrated an increase in the cytoplasmic portion of PTB (data not shown). At 32°C however, the temperature at which we have previously shown PTB to play a more apparent role in the viral life cycle in CRFK cells [34], this observed effect of FCV infection on PTB localization was more dramatic and infected cells demonstrated decreased nuclear and increased cytoplasmic PTB levels (Fig 5a). Quantification of staining intensity from 25–40 infected cells, demonstrated a noticeable correlation between the levels of the NS6-7 protein present in a cell and the extent to which PTB has been redistributed to the cytoplasm (Fig 5b and c). Increased levels of NS6-7 in the cytoplasm of the infected cell correlated with decreased levels of nuclear PTB and increasing levels of cytoplasmic PTB (Fig 5b and c). This observation indicated that as infection progresses, PTB is redistributed from the nucleus to the cytoplasm. This was further verified by examining the localization of a GFP-PTB fusion protein during the course of FCV infection using time-lapse image analysis (Fig 6a and supplementary Figure S1). CRFK cells expressing GFP-PTB were infected with a recombinant FCV expressing a LC-DsRED fusion protein as described [38]. As infection progressed, evident by the appearance of LC-DsRED, clear relocalization of GFP-PTB occurred (Fig 6a). To examine if the observed effect was a global effect on nuclear-cytoplasmic localization or a more specific effect on PTB localization, cells expressing GFP-PTB and DsRED2 fused to 3 copies of the nuclear localization sequence of the SV40 large T-antigen (DsRED2-Nuc), were infected with FCV and examined by time-lapse microscopy (Fig 6b and supplementary Figure S2). The progress of infection resulted in a gradual increase of the cytoplasmic fraction of the PTB protein starting from 7.5 hpi. Similar to the time-lapse experiments with FCV-LC-DsRED virus, a significant release of the PTB was observed ∼ at 13 hpi, 1.5 hours prior to relocalization of DsRED2-Nuc (Supplementary Figure S2).

### PTB Partially Localizes with FCV Replication Complexes and Interacts with Viral RNA during Infection

**Figure 7:**
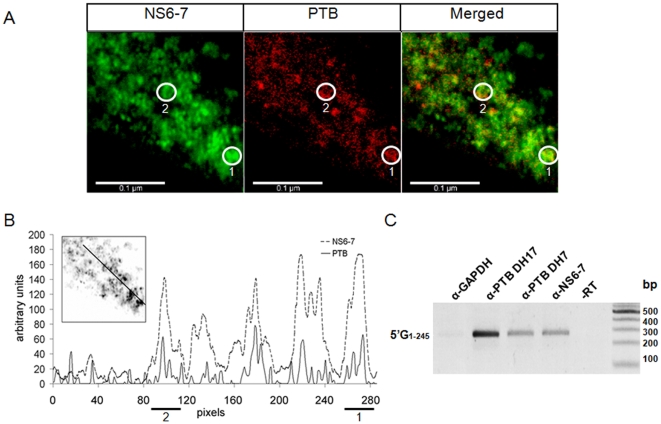
PTB colocalizes with mature FCV replication complexes in FCV infected CRFK cells. CRFK cells were infected with FCV for 16 h at 32°C and at an m.o.i. of 0.5. (A) Infected cells were fixed and stained for NS6-7 (green) and PTB (DH17 monoclonal, red). DAPI was used for nuclei (blue) staining. Note that only a fraction of the cytoplasm of an infected cell was observed using confocal microscopy. (B) Plot of pixel intensity for NS6-7 and PTB along a straight line in the above image for the visualization of the colocalization of PTB with the replication complexes (NS6-7). A line of quantification was arbitrarily drawn across the image (see insect picture for the position) and the staining intensity (arbitrary units) of NS6-7 and PTB plotted against the pixel position (from left to right). Two replication complexes which contain both NS6-7 and PTB are highlighted in A and B. (C) Viral RNA immunoprecipitation from FCV infected CRFK cells at 37°C with antibodies against PTB (DH17 and DH7) and the viral NS6-7. Antibody for GAPDH protein was used as negative control and the NS6-7 IP was PCR amplified without reverse transcription to confirm the authenticity of product (-RT). The presence of the viral RNA in immunoprecipitates was confirmed by RT-PCR amplification of nucleotide 1-245 of the FCV genome.

Feline calicivirus replication occurs within the replication complexes formed in the cytoplasm [43]. A larger magnification of the cytoplasm of infected cells indicated that PTB at least partially colocalized with the NS6-7 protein within the replication complexes (Fig 7a). Quantification of NS6-7 and PTB intensity on a linear section of the image revealed that colocalization was more apparent in regions of the cytoplasm containing higher NS6-7 intensity (presumably more mature) replication complexes, revealing a possible link between cytoplasmic PTB and replication complex maturation (see highlighted replication complex in Fig 7a and b). Alternatively, it may simply reflect limited accessibility of the mAb epitope to PTB within a proportion of the membrane bound replication complexes. Although our *in vitro* data demonstrated efficient PTB binding to the FCV genome, it was important to demonstrate that this interaction also occurred during viral infection. To address this question, RNA immunoprecipitation was used to determine if PTB was physically associated with the viral RNA during replication. Two monoclonal antibodies (DH7 and DH17) to PTB that efficiently immunoprecipitated PTB from cell extracts (data not shown) were able to co-precipitate the FCV RNA from infected CRFK cells as determined by RT-PCR (Fig 7c). A polyclonal antibody against the viral protease-polymerase, NS6-7, was used as a positive control, while a monoclonal antibody against GAPDH was unable to co-immunoprecipitate the viral RNA, confirming the specificity of the interaction (Fig 7c).

### PTB Inhibits FCV Translation *In Vitro*

**Figure 8:**
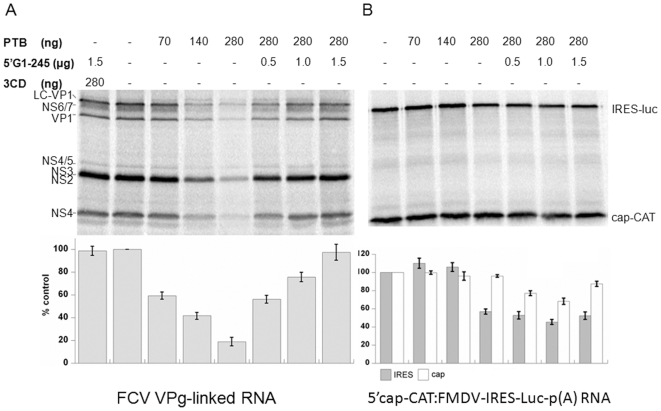
PTB inhibits FCV translation *in* vitro. (A) Effect of his-PTB on FCV *in vitro* translation that demonstrates the reduced expression of FCV proteins produced by the translation of ORF1 (from the genomic RNA) and ORF2 (from the subgenomic RNA). The ^35^S methionine labeled FCV proteins were analyzed by SDS-PAGE. Addition of increasing amounts of *in* vitro transcribed RNA encompassing nucleotides 1–245 of the FCV genome (5′G1-245) led to the recovery of the FCV translation. The bar chart illustrates the efficiency of FCV translation as percentage of the control (0 ng PTB) as measured by phosphorimager quantification. The poliovirus protease-polymerase 3CD was used to control for any non-specific effects of adding recombinant protein to the reaction. Error bars represent the standard deviation from three different experiments. (B) Increasing amounts of his-PTB and 5′G 1-245 in the *in vitro* translation of a dicistronic RNA construct that expressed the CAT enzyme under the cap structure and firefly luciferase under the FMDV IRES. The ^35^S methionine labeled CAT and firefly luciferase were analyzed by SDS-PAGE. The bar chart illustrates the efficiency of cap- and FMDV IRES-dependent translation as a percentage of the control (0ng PTB) as measured by phosphorimager quantification. Error bars represent the standard deviation from three different experiments.

In order to define the stage of the virus life cycle at which PTB might be involved, the uncoupling of the two main processes, translation and replication, was required. These two processes are inherently linked for all positive-strand RNA viruses since the viral RNA functions both as a template for replication by the RNA polymerase and as a template for translation. In addition, the unique mechanism of calicivirus VPg-dependent calicivirus translation initiation which relies on the interaction of host cell translation initiation factors with the VPg protein covalently linked to the 5′ end of viral RNA [29], [30], [31], precludes the use of *in vitro* transcribed RNA for the study of authentic virus translation and replication. However, we have recently developed an *in vitro* translation system [31], which allows some aspects of calicivirus translation initiation to be characterized in the absence of genome replication. As PTB is a well known regulator of picornavirus translation [13] its effect on FCV translation was assessed by titration of recombinant his-PTB in a rabbit reticulocyte lysate (RRL) FCV *in vitro* translation assay (Fig 8). Addition of increasing amounts of PTB resulted in a decrease in the production of *in vitro* synthesized ^35^S-labeled FCV proteins (Fig 8a), indicating that PTB may function as a negative regulator of calicivirus translation initiation. To confirm that the observed effect was specific for the interaction of PTB with the FCV genome, *in vitro* transcribed RNA encompassing the FCV genome from ntds1-245 (5′G_1-245_) was titrated back into reactions containing inhibitory levels of recombinant PTB (Fig 8a). FCV translation was restored back to WT levels when exogenous FCV 5′G_1–245_ RNA was added to the reaction, confirming the specificity of the effect. A 5′end capped and 3′ polyadenylated dicistronic RNA expressing the CAT enzyme in a cap-dependent manner and firefly luciferase under the control of the FMDV IRES (5′cap-CAT:FMDV-IRES-Luc-p(A)) was used to assess the effects of PTB on the general cap-dependent and IRES-dependent translation (Fig 8b). Upon addition of low amounts of PTB into the translation reactions cap-dependent translation was not affected, while FMDV translation demonstrated a slight increase (Fig 8b). Under the same conditions, FCV translation was reduced by 40 and 60% when 70 ng and 140 ng of PTB was added to the translation reactions respectively (Fig 8a). At the highest concentration of PTB both the FMDV IRES dependent and cap dependent translation were slightly inhibited (Fig 8b). In contrast to FCV translation, titration of the 5′G_1-245_ RNA transcript into reactions containing 280 ng of PTB, did not restore the levels of either cap-dependent or FMDV IRES-dependent translation. Therefore it is possible that the observed inhibitory effect on cap and IRES-dependent translation may be the due to contaminating factor(s) co-purified with PTB from *E.coli*, rather than PTB itself. Alternatively the FCV G_1–245_ sequence may be unable to compete for PTB binding to the dicistronic RNA or high levels of PTB may have resulted in competition for other factors required for general translation initiation in the RRL.

### FCV Translation Is Enhanced by RNAi-Mediated Knockdown of PTB

**Figure 9:**
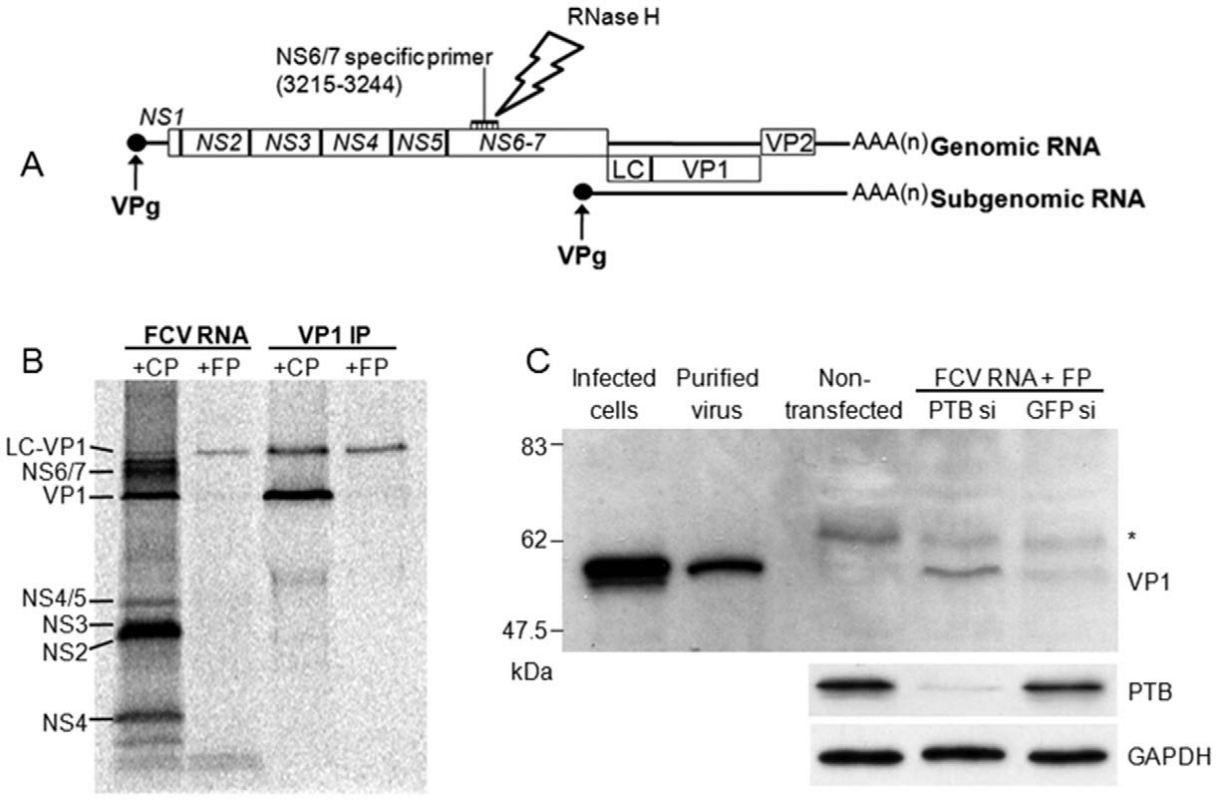
RNAi mediated knockdown of PTB inhibit the translation from the FCV subgenomic RNA in cells. (A) Genome schematic of the FCV genome highlighting the position of the three FCV open reading frames and the NS6-7 specific primer which bound to nucleotides between 3215–3244 used for RNase H-directed inactivation of the FCV genomic RNA. (B) *In vitro* translation of FCV RNA after RNase H digestion using an FCV genomic RNA specific DNA oligonucleotide (FP) and a control DNA oligonucleotide (CP). Immunoprecipitation (VP1-IP) with α-major capsid (VP1) from the *in vitro* translation of FCV RNA after RNase H digestion using an FCV genomic RNA specific DNA oligonucleotide and a control DNA oligonucleotide. The proteins were labeled with ^35^S methionine and the SDS-PAGE analysis was exposed to a phosphorimager screen. (C) Western blot for the major capsid protein (VP1) in infected CRFK cells, purified virus, non-transfected CRFK cells and CRFK cells treated with PTB or GFP siRNAs that have been transfected with FCV RNA digested with RNase H using an FCV specific DNA oligonucleotide. Control blots for PTB and GAPDH are shown to demonstrate PTB knockdown and equal loading of samples. Results displayed were obtained from cells incubated at 37°C but identical results were also observed when the experiments were performed at 32°C (data not shown) An asterisk is used to highlight a non-specific protein with reactivity to anti-VP1 antisera.

As stated above, the requirement for the covalent linkage of the virus encoded VPg protein to the 5′end of FCV RNA for efficient translation excludes the use of *in vitro* transcribed RNA for the study of authentic calicivirus translation initiation. During virus replication a sgRNA is produced from the 3′ half of the viral genomic RNA, which, similarly to the genomic RNA, is translated following the recruitment of translation initiation factors by the VPg protein. However, the sgRNA does not produce the viral polymerase and encodes only the major capsid precursor protein (LC-VP1) and the minor capsid (VP2) protein. Thus, transfection of cells with VPg-linked sgRNA alone allows the analysis of virus translation in the absence of replication as there is no source of the NS7 RNA polymerase. We have previously demonstrated that PTB also binds the 5′ end of the sgRNA [34], therefore as an attempt to separate the two major processes of the FCV life cycle, namely translation and replication, was made by specific digestion and functional inactivation of the genomic RNA within VPg-linked RNA preparations using DNA oligonucleotide-directed RNase H mediated digestion. A DNA oligonucleotide complementary to the coding region of the protease-RdRp (NS6-7) was annealed to the viral RNA preparation and the complex was digested by the DNA:RNA hybrid specific RNase H (Fig 9a). The efficiency of RNase H in removing the genomic RNA was assessed by *in vitro* translation of the digestion reaction in RRLs (Fig 9b). The viral RNA treated with a non-specific oligonucleotide produced a normal FCV protein profile, while the use of RdRp specific oligonucleotide resulted in the elimination of all the viral proteins encoded by the genomic RNA (Fig 9b). The FCV major capsid protein is produced as a premature protein (LC-VP1) attached to a leader polypeptide that is subsequently removed by the viral protease, the NS6-7 protein. The absence of NS6-7 in the protein profile is not only apparent directly by the lack of a radiolabeled protein at the correct position in the gel, but also indirectly by the fact that the LC-VP1 remained uncleaved in the *in vitro* translation reactions (Fig 9b). The identity of the bands corresponding to the LC-VP1 and the capsid in the FCV *in vitro* translation were verified by immunoprecipitation using an α-VP1 antibody (Fig 9b). The RdRp specific RNase H digested viral RNA was transfected into CRFK cells treated with PTB or GFP specific siRNAs to assess the effect of PTB knockdown on viral translation. As shown by western blot for the capsid protein, the production of protein from the sgRNA was greatly increased in the absence of PTB (Fig 9c). This suggests that siRNA-mediated knockdown of PTB reduced the proposed PTB-mediated inhibition in FCV translation resulting in increased translation from the transfected sgRNA. It is interesting to note however that the capsid protein that we detected had a similar molecular weight to the mature major capsid protein that is present in infected cells and virions (Fig 9c), while the premature LC-VP1 precursor protein, produced by the sgRNA in the absence of NS6-7, was not detected. It has been reported previously that non-caliciviral proteases are capable of processing LC-VP1 as maturation of VP1 and VLP production was observed when LC-VP1 was expressed in CRFK cells and other cell lines when co-infected with *Vaccinia* virus [44], [45]. It is also possible that the production of an apparent mature capsid cleavage product is the result of cleavage by cellular caspases, induced as the result of multiple transfections, and previously known to process the FCV capsid protein [46]. In addition, we cannot formally exclude the possibility that very low levels of NS6-7 were present due to small amount of genomic RNA not removed during RNase H treatment.

### Mutation of the PTB Binding Sites Results in a Cell Type Specific Effect on FCV Replication

**Figure 10:**
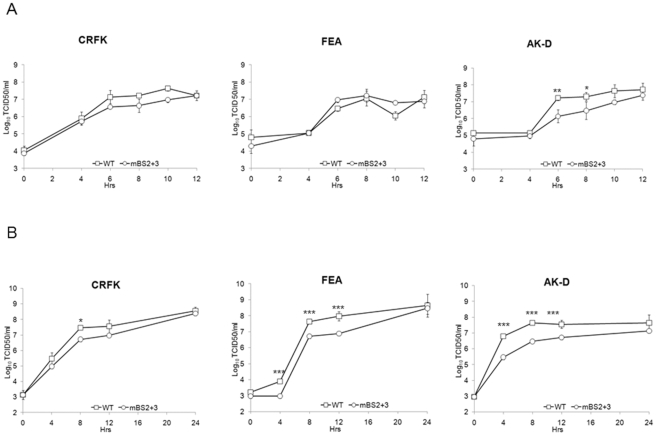
Mutations in PTB binding sites 2 and 3 results in a cell type specific defect in FCV replication. (A) Single and multi-cycle (B) growth curve analysis of wild type FCV Urbana strain derived from cDNA (WT) or a derivative carrying synonymous nucleotide changes in PTB binding sites 2 and 3 at the 5′ end of the genomic RNA. Cells were infected with a m.o.i. of 3 for single-cycle and 0.01 for multi-cycle (based on the titre of viral stocks in CRFK cells) and samples harvested at various times post inoculation. Viral titre was subsequently determined by TCID50 on CRFK cells and expressed as TCID50 per ml. Infections were performed in triplicate and the error bars represent the standard deviation. *** *P*<0.001 *P<0.01 *P<0.05 by two-way ANOVA.

**Figure 11:**
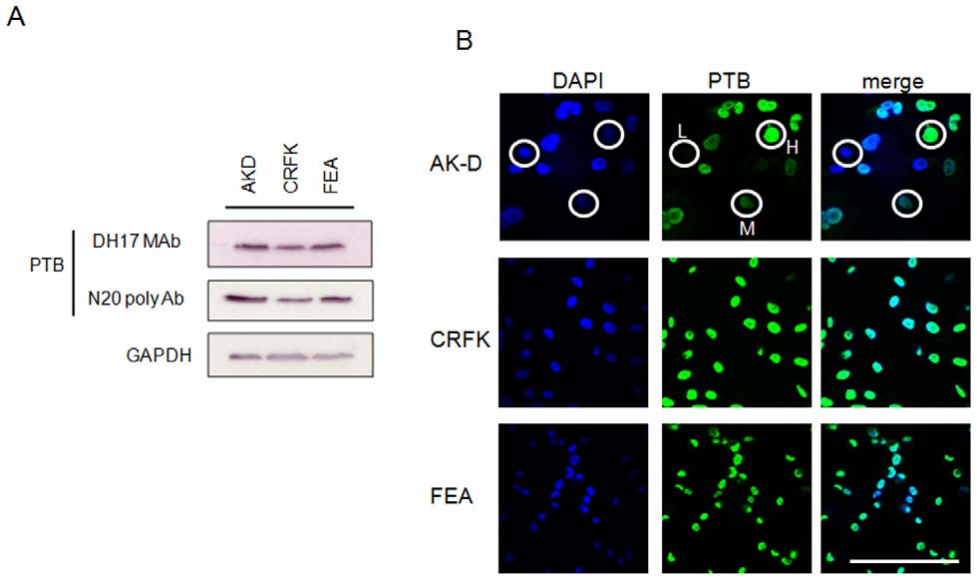
Characterisation of PTB distribution and expression levels in various cell types. (A) Western blot and (B) confocal image analysis of the expression and cellular distribution of PTB in uninfected AK-D, CRFK and FEA cells. Approximately 1×10^5^ cells were lysed and analysed by western blot using both monoclonal (DH17) and polyclonal (N20) antibodies to PTB (A). GAPDH was used to ensure equal loading. (B) Confocal analysis of AK-D, CRFK and FEA cells stained for DNA with DAPI (blue) and PTB (DH17 monoclonal, green). L, M and H highlight AK-D cells expressing low, medium and high levels of PTB respectively. Size bar represents 100 µm.

To further validate that PTB binding to the FCV genome plays a role in the FCV life cycle, we examined the effect of synonymous changes in the PTB binding sites on virus replication. Based on our observation that PTB may negatively regulate FCV translation, we expected that reduced PTB binding might lead to an inability to regulate translation and replication rates, resulting in reduced virus titre. We therefore examined the growth of wild type FCV and a strain containing synonymous mutations in BS2 and 3 within the 5′ end of the viral genomic RNA (mBS2+3) in three different cell lines; CRFK (feline kidney cell line), FEA (feline embryonic airway) and AK-D cells (feline fetal lung). The effect of mutations in the 5′ end of the genomic RNA were examined as we speculated that any alteration of the efficiency of translation and/or replication of the viral genomic RNA, which encodes the replicase components, would have a more apparent effect on virus titre than similar mutations in the viral sgRNA, which encodes the structural components only. Single step growth curve analysis indicated that the mBS2+3 virus had a cell type specific defect in virus replication, displaying reduced replication kinetics in the fetal lung cell line AK-D (Fig 10a and b). A slight reduction in mBS2+3 titers was observed in CRFK and FEA cells at 6 hours post infection as determined by titration of infectious virus produced (Fig 10a). In AK-D cells however, a greater (>10 fold) reduction in virus titre was observed when compared to the wild type virus (Fig 10a). The decrease in mutant virus replication was more apparent when low m.o.i. multi-cycle growth curves analysis were performed (Fig 10b). At 8 hours post infection mBS2+3 displayed a 5.6, 8.3 and 14.7 fold reduction in CRFK, FEA and AK-D cells respectively compared to WT virus. These data suggested that PTB binding to the 5′ end of the FCV genomic RNA is required for optimal virus replication. Our analysis also indicated that *in vitro* translation of mBS2+3 viral RNA was repressed to a lesser degree by recombinant PTB than WT viral RNA (data not shown). Hence the observed defect in replication is likely the result of an inability to coordinate translation and replication. To further elucidate the nature of the cell type specific effect and examine if there was any correlation between the observed effects of the binding site mutations and the levels of cellular PTB, we examined the steady-state levels of PTB in the various cell lines using both monoclonal and polyclonal antibodies (Fig 11a). We observed that of the three cells lines examined, CRFK cells showed the lowest levels of PTB expression, whereas AK-D and FEA cells appeared to express comparable overall levels (Fig 11a). However, further analysis by confocal imaging illustrated that whereas CRFK and FEA cells displayed a somewhat uniform expression level between the various cells within a culture, the AK-D population was very heterogeneous (Fig 11b). There were clear examples of cells which expressed high levels of PTB and also those in which PTB was undetectable (circled in Fig 11a). This may be a reflection of the cell cycle mediated regulation of PTB as PTB transcripts have been reported to be induced in the S and G2M phase of certain cell types [47]. Hence this variation in PTB levels may in some way contribute to the cell type specific effect on the growth of the mBS2+3 recombinant virus, again highlighting that PTB expression and binding plays an important role in the regulating the efficiency of virus translation and replication.

## Discussion

Our previously published data revealed that PTB was required for the efficient replication of FCV, as siRNA mediated knockdown of the protein significantly reduced the virus yield in CRFK cells [34]. During positive-strand RNA virus replication genome translation is linked to viral genome replication, hence any effects on translation have knock-on effects on replication and *vice versa*. Thus, the reduction in FCV virus replication and subsequent yield of infectious virus that we previously observed upon PTB knockdown could not be attributed to one specific function of PTB within the viral life cycle.

Previous studies have highlighted that the primary role for PTB in the life cycle of several positive-strand RNA viruses is at the level of viral translation, primarily functioning as an enhancer of translation initiation [14], [48]. It is believed that the stabilization of a functional RNA structure or conformation of the viral IRES by PTB modulates the association of the IRES with the translation machinery [49], [50]. However picornavirus protease mediated cleavage of PTB is also thought to result in the inhibition of translation, promoting a switch from translation to replication [51]. Although PTB is known to act as a translation enhancer, a recent report demonstrated a repressive role for PTB on the translation of the Oskar protein in *Drosophila*, highlighting that the action of any RNA-binding protein largely depends on the RNP that it is a component of [52]. Studies have also shown that PTB interacts with the 5′ and 3′ ends of the Norwalk virus genome and although the function of this interaction has yet to be determined [53], [54], recent work has highlighted that the 5′ and 3′ ends of the Norwalk virus genome interact via binding to host cell proteins to form an RNP [55], of which PTB may be a component.

This study was designed to try to address the fundamental role of PTB in the calicivirus life cycle using feline calicivirus as a model system. Prior to this study, the only available system to allow the examination of FCV translation, in the absence of genome replication was the *in vitro* RRL translation system [31]. Using this system we demonstrated that PTB may function as a negative regulator of FCV translation (Fig 8), as is the case for the *Drosophila* Oskar protein. During these studies we found that although at low levels of exogenous PTB, a limited increase of the FMDV IRES activity *in* vitro was observed, higher levels led to a significant reduction, presumably through the titration of additional factors. The original investigations into the involvement of PTB in FMDV translation, revealed that dependence on PTB was observed only in RRLs depleted of PTB [13]. Work on PTB depleted RRLs showed that absence of PTB did not affect FCV translation, while FMDV translation was significantly reduced (data not shown). In our previous work, PTB knockdown had a temperature-dependent effect on FCV replication, which may reflect a higher requirement for the RNA chaperone activity of PTB at temperatures where RNA folding is sub-optimal [34]. Thus, we also assessed how the inhibitory effect of PTB varied at different temperatures, despite the fact that the optimal temperature range for *in vitro* translation is significantly lower than the temperature for the normal virus infection. The inhibitory effect of PTB was more apparent at temperatures lower or higher than the optimal 30°C (data not shown) providing additional evidence towards the RNA chaperone activity of PTB under suboptimal conditions.

Due to the unique mechanism of calicivirus translation, an attempt to verify the role of PTB in FCV translation in cell culture was made by selective digestion of the genomic RNA in the mixture of viral RNAs using RNase H. The inactivation of the genomic RNA allowed only translation from the sgRNA that, in the absence of polymerase, cannot be replicated. It is known that both genomic and subgenomic RNA are translated by the same VPg-dependent mechanism [29] and given our previous observations that PTB also binds the sgRNA [34] we examined the effect of PTB knockdown on viral RNA translation in the absence of replication. The translation of the sgRNA in CRFK cells, as determined by VP1 expression, was significantly increased under low levels of PTB (Fig 9), confirming a repressive role of PTB on viral translation in cells. Surprisingly our results indicated that the capsid precursor protein LC-VP1 was processed into the mature form of VP1 (Fig 9c). Previous reports have highlighted that infection of cells with *Vaccinia* virus can result in the processing of the FCV LC-VP1 into the mature VP1 protein [44], [45]. This may have been either due to a *Vaccinia* virus encoded protease or the induction of a cellular stress leading to the expression of a protease responsible for the observed processing. It is therefore possible that the multiple transfections required to obtain efficient PTB knockdown, followed by subsequent transfection with viral sgRNA also resulted in the induction of a similar cellular stress related protease which processed LC-VP1 to produce a protein of approximately the same mass as the mature capsid protein. Indeed previous studies have highlighted that caspase 2 induced during FCV infection can cleave the mature capsid protein (60kDa) during FCV infection to produce a ∼40kDa product [46]. Therefore caspase mediated cleavage of the premature protein (73kDa) might produce a ∼53kDa product, which may in fact be the product observed during our studies (Fig 9c). It is also possible that whilst we were unable to detect any NS6-7 protease in cells transfected with the sgRNA preparations (data not shown), we cannot rule out that a very small amount of this highly processive enzyme was expressed from low levels of viral genomic RNA not removed by the RNase H digestion. Despite this, the interpretation of our data i.e. that PTB knockdown stimulates viral translation, remains valid.

At first glance the increased levels of viral sgRNA translation in PTB depleted CRFK cells is contrary to our previously observed reduced viral replication (observed at both protein and viral RNA levels) [34]. However, it is important to note that the genomes of positive-strand RNA viruses functions for both translation and replication, hence any interference with one step will have a concomitant effect on the other. It has previously been demonstrated that actively translating ribosomes can efficiently inhibit poliovirus RNA synthesis [56] and that the interaction of the viral polymerase precursor 3CD with the 5′ end, in combination with the cellular protein PCBP2, inhibits translation promoting negative strand RNA synthesis [57]. At the initial stages of infection it is crucial for the infecting virus to transform the intracellular environment in order to achieve efficient replication [43]. This occurs by translation of the viral RNA to produce the proteins required for the rearrangement of the cellular membranous structures which lead to the formation of the replication complexes [58]. As the microenvironment of each replication complex becomes saturated with viral proteins, translation is decelerated to favor the production of new viral RNAs. This switch between translation and replication must occur as the RdRp proceeds in a direction opposite to the ribosome during negative-strand RNA synthesis. The involvement of PCBP2 in the switch between translation and replication in polioviruses highlights the importance of host-cell RNA binding proteins in this process [57]. It is possible that PTB, through interaction with the calicivirus RNA, and possibly in combination with other viral or cellular proteins, PTB decreases the translation rate by sequestration of the 5′ extremity from the translational machinery. The result is the formation of an RNP, of which PTB is a component, which promotes replication at the later stages of the virus life cycle. Cleavage of PTB and PABP has also been suggested as possible mechanisms of controlling the switch between picornavirus genome translation and replication [51], [59]. Our analysis would indicate that no stable PTB cleavage products are visible in FCV infected cells [34], however the previously described cleavage of PABP mediated by both Norwalk and FCV protease may also contribute to any potential switch in translation and replication [60]. It is worth noting however that although calicivirus genomes possess a poly(A) tail, a direct role for PABP in calicivirus translation has yet to be demonstrated. In addition, PABP cleavage was observed only at late stages of FCV infection, where most viral replication has already taken place and viral titres approach their maximal level (see Fig 10a). It is therefore likely that PABP cleavage may serve primarily to reduce host cell protein synthesis, promoting cell death and virus release, although clearly further studies to address this are warranted.

As PTB is predominantly nuclear, our observed cytoplasmic relocalization during FCV infection would add to our hypothesis on the involvement of PTB in the maturation of the viral replication complexes. It is noteworthy that only partial colocalization of PTB with NS6-7 was observed during our study (Fig 7a). Whilst this may indicate that much of the NS6-7 is not actively engaged in viral genome replication, it may also indicate that PTB is present only in “mature” replication complexes which are no longer translating viral genomes but are actively engaged in viral genome replication. In addition, we cannot rule out the possibility that this lack of complete colocalization is not simply the result of lack of accessibility of the PTB mAb epitope within many of the membrane bound replication complexes. Our real-time analysis of the effect of virus infection, clearly demonstrated that PTB redistribution to the cytoplasm occurs prior to any global effect on nuclear-cytoplasmic shuttling (Fig 6). It is now well established that nuclear factors important for viral replication, such as PTB, translocate from the nucleus to the cytoplasm during poliovirus [7], rhinovirus [61] and dengue virus [6] infection. In some cases the mechanism of this relocation has been linked to an observed modification of the components of the nuclear pore [7], [61]. The observation that, in addition to the specific effect on PTB localization, FCV infection also has a more general effect on the import of proteins containing an NLS (Fig 6b), may indicate that FCV also modifies the nuclear pore in some way. It is possible that virus induced apoptosis is responsible as caspases have been reported to cleave nucleporins [62], although whether this occurs during FCV infection will require further verification.

The cell type specific effect of the PTB binding site mutations is somewhat intriguing and may be due to the apparent heterogeneity of PTB expression in AK-D cells (Fig 11b). Previous reports have highlighted that PTB transcripts can to be induced in the S and G2M phase of certain cell types [47] but whether this is the case in AK-D cells remains to be determined. It is well established that variations in the expression of PTB and PTB isoforms can result in cell type specific effects on RNA viruses [63]. It is also possible that in addition to PTB, the cell type specific variation in expression of other, as yet unidentified cellular factors which function as part of the PTB-containing RNP, also contributes to the observed effect on FCV replication. The identification of these factors is currently the focus of ongoing studies.

**Figure 12:**
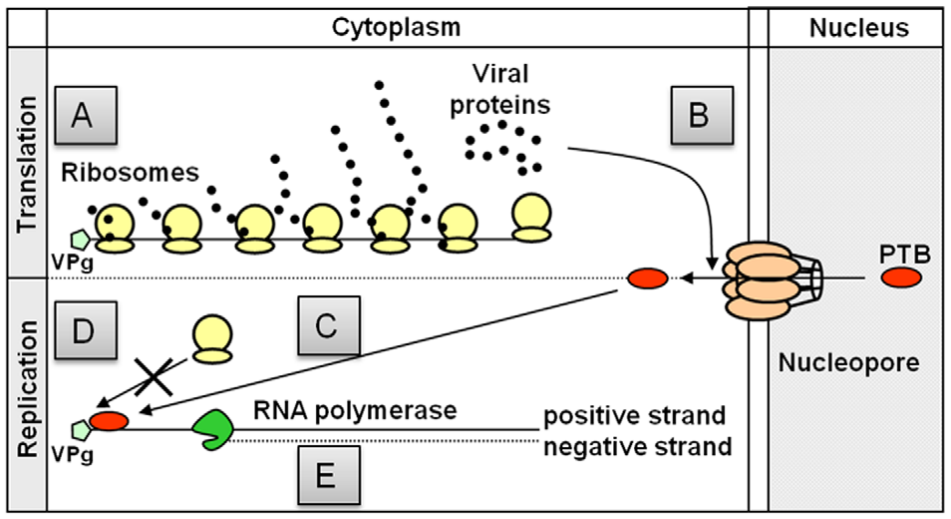
Proposed model for the function of PTB in the FCV life cycle. Schematic representation of the potential regulatory function of PTB during the various stages of the FCV infectious cycle. (A) The viral RNA is translated via VPg-dependent translation to produce the various viral proteins. (B) As the viral proteins accumulate, they modulate (directly or indirectly) the nuclear-cytoplasmic shuttling of PTB. (C) Cytoplasmic PTB interacts with the viral RNA, possibly in combination with other cellular or viral proteins to subsequently inhibit the recruitment of the ribosomes to the 5′ end of the positive-strand viral RNA (D). Displacement of the ribosomes allows the synthesis of negative strand RNA (E) by the RNA-dependent RNA polymerase.

In light of these data, we propose a speculative model for the function of PTB in the FCV life cycle detailed in figure 12. During the early stages of FCV infection translation occurs in the cytoplasm of infected cells producing the various viral proteins (Fig 12a). As the levels of viral proteins accumulate, a specific modification of the nuclear-cytoplasmic shuttling of PTB occurs (Fig 12b) causing PTB to translocate from the nucleus to the cytoplasm (Fig 12c) to form an RNP complex at the 5′ end of the viral RNA (Fig 12d). PTB binding to the 5′ end, probably in combination with other host cell or viral factors, prevents translation initiation and ribosome recruitment (Fig 12d) promoting the synthesis of viral RNA (Fig 12e). The above findings support a role for the first RNA-binding protein to be identified as part of a functional calicivirus RNP complex and give further insight in the molecular mechanism used for genome translation and replication by this important group of pathogens.

## Supporting Information

Figure S1 Time-lapse analysis of the effect of FCV infection on PTB localization. GFP-PTB expressing CRFK cells were infected with recombinant FCV expressing a LC-dsRED fusion protein (m.o.i. 2) and examined during the course of the infection. XVID file.
(1.01 MB AVI)

Figure S2 Time-lapse analysis of the effect of FCV infection on nuclear localization. Cells transfected with cDNA constructs expressing PTB-GFP and dsRED fused to 3 copies of the nuclear localization sequence of the SV40 large T-antigen (DsRED2-Nuc) were infected with FCV at a m.o.i. of 2. Cells were then examined over the course of the infection. XVID File.
(1.38 MB AVI)
